# Dental characteristics of patients with four different types of skeletal dysplasias

**DOI:** 10.1007/s00784-023-05194-w

**Published:** 2023-08-07

**Authors:** Worasap Tantibhaedhyangkul, Jenjira Tantrapornpong, Nuttanun Yutchawit, Thanakorn Theerapanon, Narin Intarak, Sermporn Thaweesapphithak, Thantrira Porntaveetus, Vorasuk Shotelersuk

**Affiliations:** 1 Center of Excellence in Genomics and Precision Dentistry, Department of Physiology, Faculty of Dentistry, Chulalongkorn University, Bangkok, 10330 Thailand; 2 Department of Prosthodontics, Faculty of Dentistry, Chulalongkorn University, Bangkok, 10330 Thailand; 3 Center of Excellence for Medical Genomics, Department of Pediatrics, Faculty of Medicine, Chulalongkorn University, Bangkok, 10330 Thailand; 4 Excellence Center for Genomics and Precision Medicine, King Chulalongkorn Memorial Hospital, the Thai Red Cross Society, Bangkok, 10330 Thailand

**Keywords:** Achondroplasia, Acromicric dysplasia, Bone dysplasia, Cleidocranial dysplasia, Hypochondroplasia, Hypophosphatasia

## Abstract

**Objective:**

Skeletal dysplasia (SD) comprises more than 450 separate disorders. We hypothesized that their dental features would be distinctive and investigated the tooth characteristics of four patients with different SDs.

**Material and methods:**

Four SD patients with molecularly confirmed diagnoses, Pt-1 acromicric dysplasia, Pt-2 hypophosphatasia and hypochondroplasia, Pt-3 cleidocranial dysplasia, and Pt-4 achondroplasia, were recruited. A tooth from each patient was evaluated for mineral density (micro-computerized tomography), surface roughness (surface profilometer), microhardness, mineral contents (energy-dispersive X-ray), and ultrastructure (scanning electron microscopy and histology), and compared with three tooth-type matched controls.

**Results:**

Pt-1 and Pt-3 had several unerupted teeth. Pt-2 had an intact-root-exfoliated tooth at 2 years old. The lingual surfaces of the patients’ teeth were significantly smoother, while their buccal surfaces were rougher, than controls, except for Pt-1’s buccal surface. The patients’ teeth exhibited deep grooves around the enamel prisms and rough intertubular dentin. Pt-3 demonstrated a flat dentinoenamel junction and Pt-2 had an enlarged pulp, barely detectable cementum layer, and ill-defined cemento-dentinal junction. Reduced microhardnesses in enamel, dentin, and both layers were observed in Pt-3, Pt-4, and Pt-1, respectively. Pt-1 showed reduced Ca/P ratio in dentin, while both enamel and dentin of Pt-2 and Pt-3 showed reduced Ca/P ratio.

**Conclusion:**

Each SD has distinctive dental characteristics with changes in surface roughness, ultrastructure, and mineral composition of dental hard tissues.

**Clinical relevance:**

In this era of precision dentistry, identifying the specific potential dental problems for each patient with SD would help personalize dental management guidelines.

**Supplementary Information:**

The online version contains supplementary material available at 10.1007/s00784-023-05194-w.

## Introduction

Skeletal dysplasias (SDs) are a group of more than 450 inherited bone and cartilage disorders, including cleidocranial dysplasia (CCD), acromicric dysplasia (ACMICD), osteogenesis imperfecta (OI), hypophosphatasia (HPP), achondroplasia (ACH), and hypochondroplasia (HCH). Patients with SD typically have skeletal defects, such as disproportionate limbs, curved and fragile bones, a wide fontanelle, or deviated craniofacial structures [1]. The skeletal disorders are caused by variations in genes related to bone and cartilage formation, including *RUNX2*, *LTBP3*, *FBN1*, *ALPL*, or *FGFR3*. Some of these genes are also involved in odontogenesis. *RUNX2*, which regulates osteoblastic differentiation and bone maturation, controls epithelial-mesenchymal interactions during tooth development related to tooth eruption, number, and hard tissue structure [2]. Mutations in *RUNX2* lead to CCD, which manifests as hypoplastic clavicles, delayed/unclosed skeletal sutures, retention of primary teeth, supernumerary teeth, and unerupted teeth [3]. *FGFR3* plays a crucial role in the normal development of the skeletal system and teeth. Mutations in *FGFR3* have been associated with various SDs, including ACH, HCH, thanatophoric dysplasia, Muenke syndrome, and ACH with developmental delay and acanthosis nigricans (SADDAN) [4]. Malocclusion and delayed tooth development have been reported in patients with *FGFR3* mutations [5, 6]. The *Ltbp3* gene is expressed during the cap stage of mouse tooth development [7]. *Ltbp3* mutant mice had very thin or no tooth enamel [7]. In humans, the heterozygous variants in *LTBP3* cause geleophysic dysplasia and acromicric dysplasia, while the homozygous variants are associated with amelogenesis imperfecta and short stature. HPP is characterized by reduced activity of tissue-nonspecific alkaline phosphatase (TNAP) and typically results in premature tooth loss attributable to disturbed cementum formation [8]. The absence of acellular cementum has been observed in individuals with HPP and CCD [9, 10].

In patients with OI, dentinogenesis imperfecta (DGI), reduced dentin hardness, disorganized dentinal tubules and collagen fibers, and an irregular dentinoenamel junction (DEJ) have been reported [11–14]. Several studies have reported the microhardness values of primary and permanent teeth, ranging from approximately 300 to 450 Knoop hardness in enamel and 45 to 80 in dentin [15–17]. The surface roughness values of enamel have been reported as 0.21 microns in primary teeth and 0.25 microns in permanent teeth [18]. The reported calcium to phosphorus ratio varies [17, 19]. Although a number of studies have reported the clinical and genetic features of SD, there is limited knowledge about the tooth characteristics in SD patients.

In this study, we performed a comprehensive investigation of the dental phenotypes of four patients diagnosed with SD comprising ACMICD, combined HPP and HCH (HPP + HCH), CCD, and ACH. Tooth samples were characterized by their mineral density, surface roughness, microhardness, mineral composition, ultrastructure, and histology. The results from each SD tooth sample were compared with those from three tooth-type matched controls obtained from healthy individuals.

## Materials and methods

### Clinical and genetic examinations

Four SD patients were enrolled in this study. Each patient was diagnosed with CCD, ACMICD, HPP + HCH, or ACH. Clinical and/or radiographic orodental examinations were conducted by the experience dentist (T.P.) at the Faculty of Dentistry, Chulalongkorn University. Written informed consents were obtained from the patients or their parents. The study was approved by the Research Ethics Committee (HREC-DCU 2020–059), Faculty of Dentistry, Chulalongkorn University, and performed following the ethical principles of the Declaration of Helsinki.

Genomic DNA extracted from 3 ml of peripheral blood leukocytes of the probands and their available family members underwent exome or Sanger sequencing. The variant causing ACH was detected using the *FGFR3* primers: F: CTCTCCTGTGGCTCTGGTGT and R: ACATGGTGAGCAGAGACGAG. The pathogenic variants causing ACMICD, HPP + HCH, and CCD were identified in our previous studies [3, 20, 21]. Briefly, for exome sequencing, genomic DNA was captured using a SureSelect Human All Exon version 4 kit (Agilent Technologies, Santa Clara, CA, USA) and sequenced using Hiseq 2000 Sequencer (Macrogen, Seoul, South Korea). The sequence reads were aligned to the human genome reference sequence (University of California Santa Cruz (UCSC) hg19) using the Burrows-Wheeler Alignment software (http://bio-bwa.sourceforge.net/). Downstream processing was performed with SAMtools (http://samtools.source forge. net/) and annotated by the dbSNP and 1000 genomes. Subsequently, the variants were filtered out if their frequency > 1% in the 1000 Genomes Project, Genome Aggregation Database (gnomAD), and our in-house database of 2166 Thai exomes.

### Examinations of tooth characteristics, surface roughness, and mineral density

A tooth indicated for extraction or an exfoliated deciduous tooth was collected from each patient. All teeth were preserved under similar condition, specifically in 10% formalin solution and stored at a temperature of 4 °C for a period of 2–3 weeks until further investigations were conducted. We obtained the permanent lower third molar from the ACMICD patient, the deciduous lower central incisor from the HPP + HCH patient, the extra upper premolar from the CCD patient, and the deciduous upper first molar from the ACH patient. Each patient’s tooth sample was evaluated and compared with three tooth type-matched controls collected from three unrelated healthy individuals.

To determine the surface roughness on the buccal and lingual surfaces of each tooth, a Talyscan 150 surface profilometer (Taylor Hobson) was utilized. Each surface underwent 30 measurements within a 2 × 2 mm^2^ tracing area located at the center of the surfaces, taken at intervals of 60 μm along the *Y*-axis. The stylus speed and the cut-off length were set at 1000 μm/s and 0.025 mm, respectively.

To evaluate mineral density, the samples were scanned with a micro-computerized tomographic system (μCT 35, SCANCO Medical AG). The imaging resolution was set to 30 μm voxel size. The acquired raw data was processed using Image Processing Language (SCANCO Medical AG) and evaluated for mineral density.

### Microhardness measurements

Each tooth sample was sectioned in a bucco-lingual plane parallel to its long axis using a slow-speed precision saw (Isomet 1000 Precision Saw, Buehler) equipped with a diamond disc. The sectioning process was conducted at 450 rpm and under constant water cooling. Two slices, each with a thickness of 1 mm, were carefully chosen from the middle portion of the tooth. Subsequently, the obtained tooth slices underwent grinding using 1200 grit silicon-carbide paper under water cooling, followed by polishing with alumina powder. One slice was selected for histological examination, while the other slice was subjected for microhardness assessment. The latter slice was further halved longitudinally, with one-half used for scanning electron microscope (SEM) analysis and the other half for energy-dispersive x-ray (EDX) examination.

To assess microhardness, the tooth indentations were created using a microhardness tester (FM700e Type D, FUTURE-TECH CORP.) applying a load of 100-g force (gf) for a duration of 15 s. The microhardness was measured from corresponding locations in the enamel or dentin of all samples collected from both patients and controls. Five spots in the enamel and five spots in the dentin of the crowns were measured: two on the buccal/labial side (top and bottom), one on the mid-occlusal or mid-incisal, and two on the lingual side (top and bottom). The enamel measurement site was positioned halfway between the tooth surface and the DEJ, while the dentin measurement site was situated near the DEJ. The indentation length was observed. The Knoop microhardness values were calculated using the formula: Knoop hardness value = 14,229 (test load (N)/indentation length^2^ (micron)^2^).

### Analysis of mineral composition

The tooth sections were coated with gold powder and the surface energy-dispersive x-ray (EDX) analysis was conducted using the ISIS 300 EDX-system (Oxford Instruments) to determine the elemental levels of carbon, oxygen, phosphorus (P), and calcium (Ca). All samples acquired from both the patients and controls were examined in a consistent manner. Five spots in the enamel and five spots in the dentin of the tooth crowns were measured: two in the top half, one in the middle, and two in the cervical half. The enamel measurement site was positioned halfway between the tooth surface and the DEJ, while the dentin measurement site was situated near the DEJ. The amounts of calcium and phosphorus (% weight) were calculated and presented as Ca/P ratios.

### Ultrastructural examinations

To investigate tooth surface morphology and structure, the tooth sections were etched with 37.5% orthophosphoric acid, rinsed with deionized water, and fixed overnight with 2.5% glutaraldehyde. After fixation, the samples were washed twice in phosphate-buffered saline, dehydrated in a graded ethanol series, coated with gold powder, and scanned with the scanning electron microscope (SEM, QuantaFeg 250, FEI Company).

For the histological examination, tooth slices were initially fixed in 10% neutral buffered formalin. Subsequently, they underwent a decalcification process using ethylenediaminetetraacetic acid (EDTA) for a period of 7 days. Following decalcification, the slices were dehydrated using a graded series of ethanol and embedded in paraffin. The paraffin-embedded blocks were then sectioned at a thickness of 5 µm and subjected to hematoxylin and eosin and Masson’s trichrome staining. The stained sections were examined using a light microscope (DM2000 Microscope with LAS v4.12 program, Leica).

### Statistical analysis

The surface roughness, microhardness, and EDX data were processed using IBM® SPSS® Statistics software. The differences between the SD samples and controls in each parameter were tested for significance using the independent samples *t*-test for normally distributed data or the Mann–Whitney U test for data with non-normal distribution. The tests were analyzed at a significance level of 0.05 (*P < 0.05, **P < 0.01, and ***P < 0.001).

## Results

### Clinical and genetic examinations

**Fig. 1:**
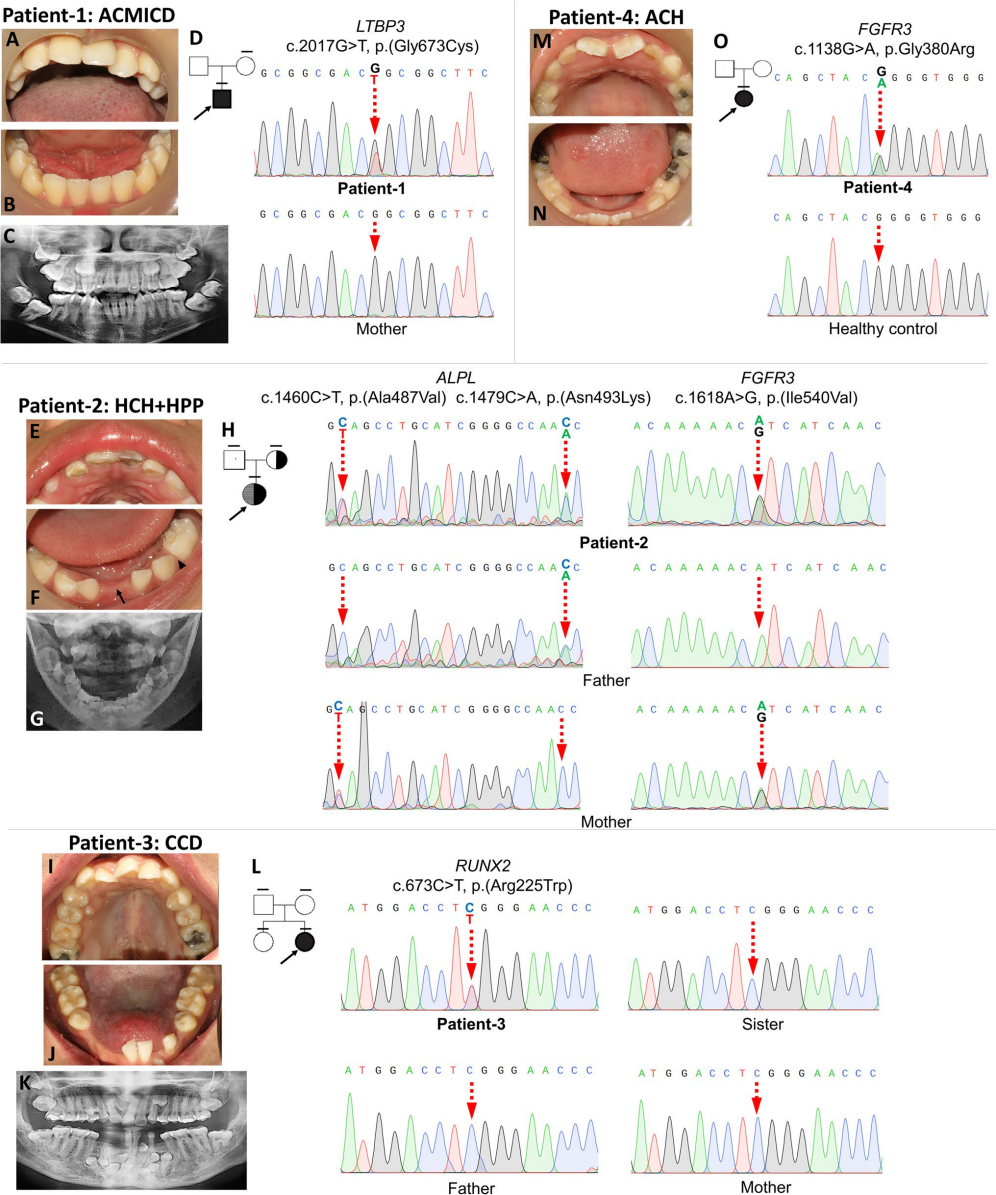
Phenotypes, family pedigree, and genotypes of SD patients. (A–D) Patient-1, a 16-year-old male with ACMICD, had microstomia, malocclusion, and unerupted teeth. The heterozygous missense *LTBP3* variant, c.2017G > T, p.(Gly673Cys), was identified in patient-1, but not in the mother. (E–H) Patient-2, a 2-year-old girl with HCH and HPP, had hypomineralized enamel, a missing deciduous lower left canine (arrowhead in (F)), and early loss of the deciduous mandibular right central incisor (arrow in (F)). The patient had the heterozygous *FGFR3* variant, c.1612A > G, p.(Ile538Val), inherited from the mother and the compound heterozygous *ALPL* variants, c.1460C > T, p.(Ala487Val) and c.1479C > A, p.(Asn493Lys), inherited from the mother and father, respectively. (I–L) Patient-3, a 20-year-old female with CCD, had multiple unerupted teeth, supernumerary teeth, retained deciduous teeth, and the *RUNX2* c.673C > T, p.(Arg225Trp) variant. Other family members did not have the variants. (M–O) Patient-4, a 7-year-old girl with ACH, had malocclusion, hypoplastic teeth, and the heterozygous *FGFR3* c.1138G > A, p.Gly380Arg variant. In the pedigree, black arrow indicates the proband. A horizontal line above each symbol indicates the subjects having genetic analyses

Patient-1, a 16-year-old male, was diagnosed with ACMICD. He had a short stature, short extremities, mild bowed legs, round face, bulbous nose, long philtrum, and thickened lips. The clinical and radiographic oral examination revealed the presence of microstomia, high-arched palate, tooth crowding, and ten unerupted teeth, including four second molars, four third molars, and two upper second premolars (Fig. 1A–C). The patient possessed the heterozygous missense variant, c.2017G > T, p.(Gly673Cys), in the *LTBP3* gene (ClinVar VCV001327506). The variant was not detected in his unaffected mother (Fig. 1D). The patient’s details were reported in our prior study [20].

Patient-2, a 2-year-old girl, was diagnosed with HCH and HPP. The patient had short upper and lower limbs, bilaterally curved legs, pretibial skin dimples, hyperlordosis, abnormal gait, prominent forehead, and midfacial hypoplasia. Her radiographs demonstrated an abnormal curvature of the long bone extremities, metaphyseal flares, and tongues of radiolucency projecting from the growth plates into the metaphyses. The patient presented with generalized enamel hypomineralization, dental caries, and the absence of the deciduous lower left canine. The deciduous upper left central incisor exhibited discoloration. Additionally, her deciduous mandibular right central incisor was exfoliated with an intact root at the age of 26 months (Fig. 1E–G). The patient harbored the heterozygous missense variant, c.1612A > G, p.(Ile538Val), in *FGFR3* (VCV000016345) inherited from the mother, and the compound heterozygous variants, c.1460C > T, p.(Ala487Val) and c.1479C > A, p.(Asn493Lys) in *ALPL* (VCV000556895, VCV001327508), inherited from the mother and father, respectively (Fig. 1H). The patient’s details were reported in our previous study [21].

Patient-3, a 20-year-old female, was diagnosed with CCD [3]. The patient had aplastic clavicles, maxillary hypoplasia, frontal and parietal bossing, Wormian bones, and widened cranial sutures. The oral examination revealed the presence of malocclusion, two retained deciduous teeth, and a total of twenty unerupted teeth, including eight supernumerary teeth (three in the maxillary arch and five in the mandibular anterior region) (Fig. 1I–K). She possessed the missense mutation, c.673C > T, p.(Arg225Trp), in *RUNX2* (VCV000009303). The p.(Arg225Trp) had been reported in both familial and sporadic CCD cases [22–24]. The variant was not detected in her unaffected parents and sister (Fig. 1L). The patient’s details were previously reported in our study [3].

Patient-4, a 7-year-old girl, was diagnosed with ACH. She presented with short stature, short limbs, broad and short hands and feet, frontal bossing, midface hypoplasia, and lumbar lordosis. The patient, who was in the mixed dentition stage, presented with malalignment of the permanent upper incisors and hypoplastic teeth (Fig. 1M, N). She was identified with the heterozygous missense variant, c.1138G > A, p.Gly380Arg, in *FGFR3* (VCV000016327) (Fig. 1O). The *FGFR3* p.Gly380Arg is a common variant associated with ACH [25, 26].

### Examinations of tooth characteristics, surface roughness, and mineral density

**Fig. 2:**
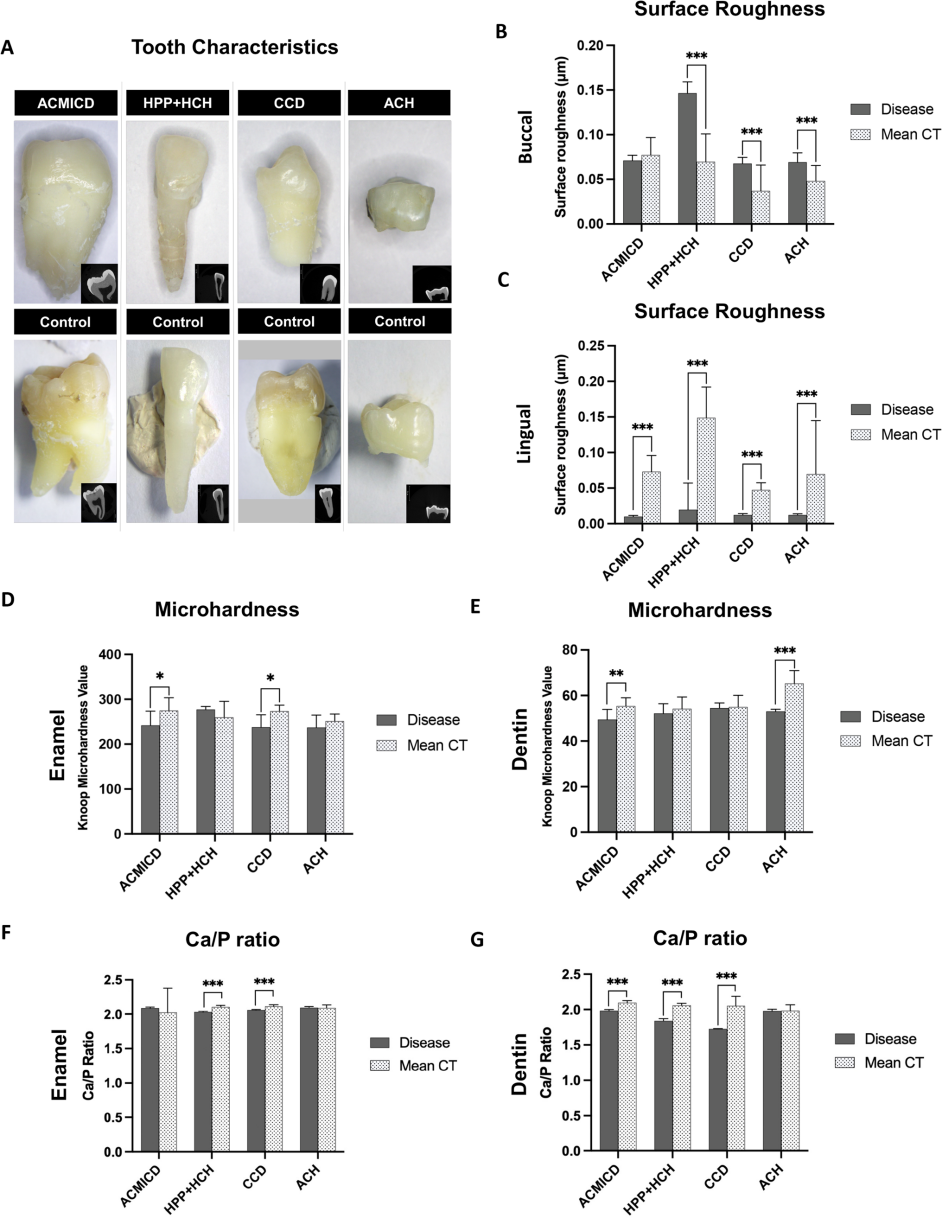
Physical characteristics of SD teeth. **A** Photographs and micro-CT images of the patient teeth revealed that the HPP + HCH tooth had hypomineralized enamel, dark-colored root, and an enlarged pulp cavity. **B**, **C** The HPP + HCH, CCD, and ACH teeth demonstrated significantly higher roughness on the buccal side, and all SD teeth showed lower roughness on the lingual side, compared with controls. **D**, **E** ACMICD and CCD showed significantly lower enamel microhardness values, while ACMICD and ACH exhibited significantly lower dentin microhardness values compared to their respective controls. **F**, **G** HPP + HCH and CCD exhibited significantly lower Ca/P ratios in both enamel and dentin, while ACMICD showed a significantly lower Ca/P ratio in dentin compared to their respective controls. *P value ≤ 0.05. **P ≤ 0.01. *** P ≤ 0.001. Ca, calcium; P, phosphorus; CT, controls

The crown appearances of the SD teeth were similar to those of controls. The HPP + HCH tooth root was darker, and its pulp cavity was larger than controls (Fig. 2A). The surface roughness values of all SD teeth were higher on the buccal sides and lower on the lingual sides, compared with those of controls, except for the buccal side of the ACMICD tooth that was not significantly different from controls (Fig. 2C). The mineral density values of the SD teeth, measured by micro-CT, were not significantly different from those in the controls (Fig. S1, Table S1).

### Microhardness measurements

The ACMICD tooth demonstrated lower enamel and dentin microhardness compared with controls. Significantly lower microhardness values were also observed in the CCD enamel and ACH dentin compared with those in controls. The HPP + HCH enamel and dentin microhardness values were comparable to those of controls. No SD teeth had higher microhardness values than controls (Fig. 2D, E).

### Analysis of mineral composition

HPP + HCH and CCD teeth exhibited significantly lower Ca/P ratios in both enamel and dentin, while ACMICD showed a significantly lower Ca/P ratio in dentin compared to their respective controls. ACH tooth did not show any significant differences in Ca/P ratios in either enamel or dentin compared with their controls (Fig. 2F, G).

### Ultrastructure examinations

**Fig. 3:**
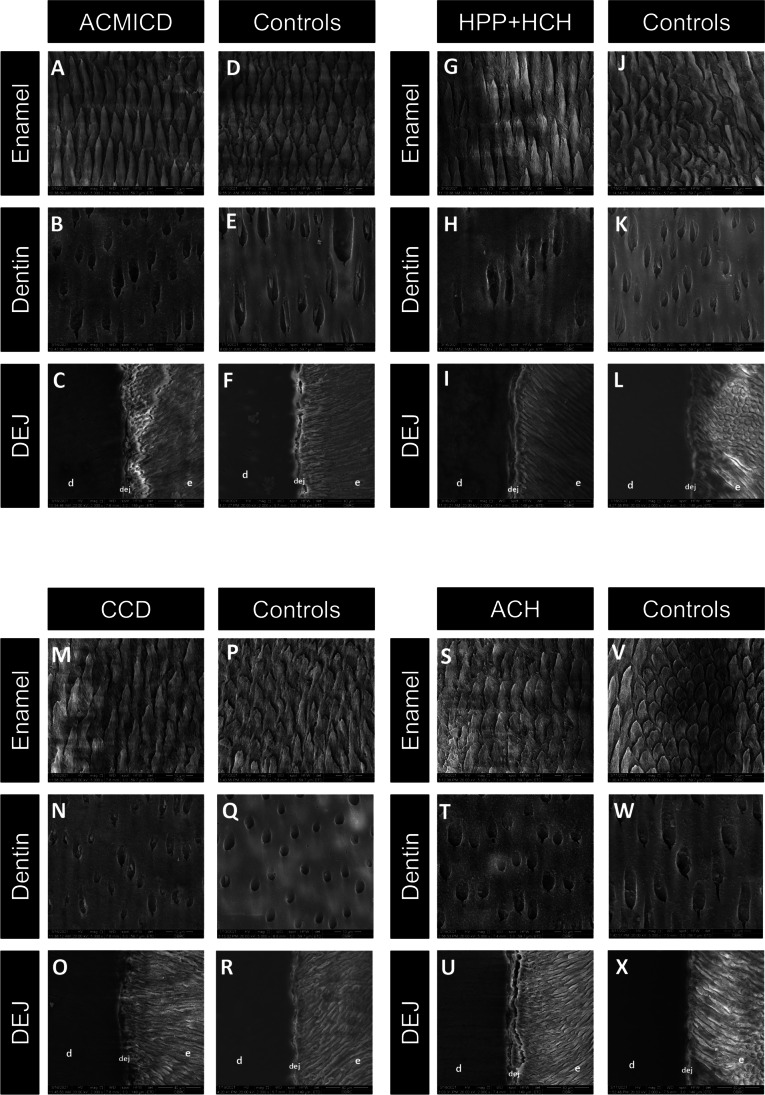
SEM of SD teeth. (A–X) Deep grooves around the enamel rods and rough intertubular dentin were observed in SD teeth. The dentinoenamel junction (DEJ) of CCD was flatter than that of controls. The DEJ of other SD teeth were comparable to those of controls. Enamel and dentin images were taken at × 5000, and DEJ at × 2000. d, dentin; e, enamel

**Fig. 4:**
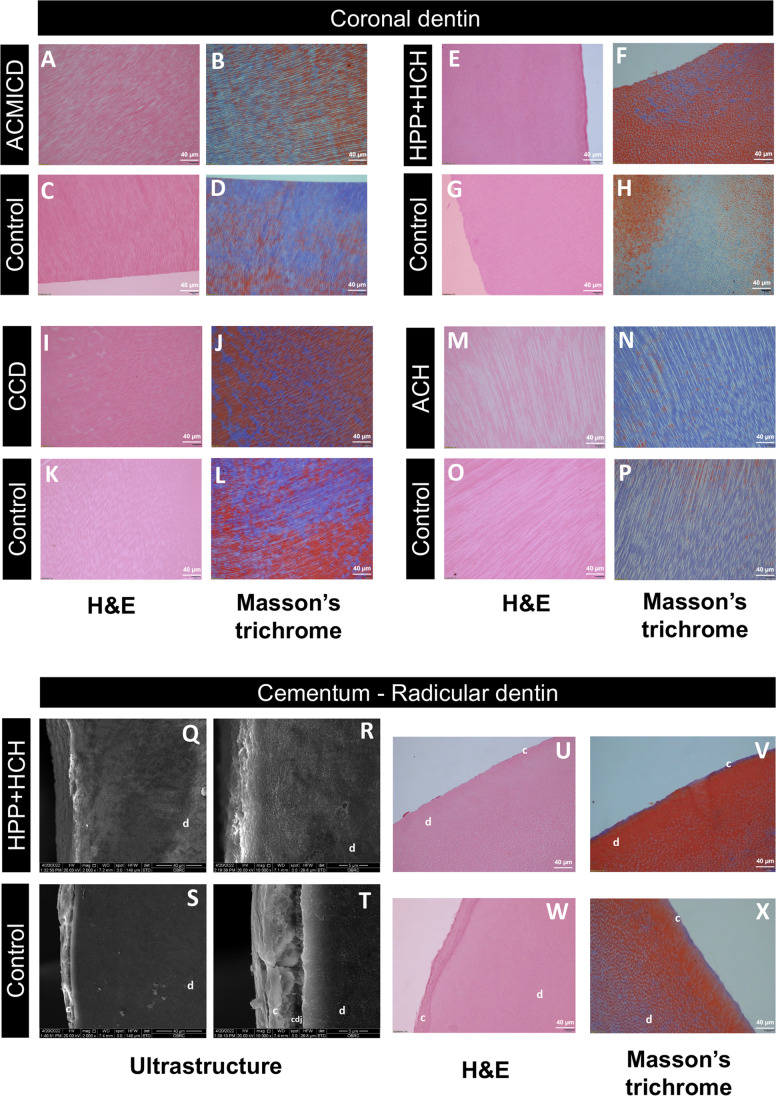
Histological analysis. (A–P) SD teeth had a dentinal tubule arrangement comparable to controls. (Q–X) A well-defined cementum layer and cementodentinal junction (CDJ) were observed in control roots (S, T, W, X), but not in HPP + HCH roots (Q, R, U V). Hematoxylin and eosin staining (× 20): A, C, E, G, I, K, M, O, U, W; Masson’s trichrome (× 20): B, D, F, H, J, L, N, P, V, X; SEM of cementum and radicular dentin: Q-T. d, dentin; c, cementum

The SEM images showed that the SD teeth exhibited deeper grooves around the enamel rods and rougher intertubular dentin surfaces, when compared to the control teeth. The DEJ of the SD teeth appeared comparable to that of the controls (Fig. 3). Histologically, the arrangement of dentinal tubules and collagen fibers in the dentin of SD teeth exhibited similarities to those observed in control teeth (Fig. 4A–P). Regarding the tooth root, SEM showed distinct differences between the control tooth and the HPP + HCH tooth. In the control, a continuous layer of cementum and a well-defined cemento-dentinal junction (CDJ) were observed. Conversely, the HPP + HCH tooth exhibited an indistinct CDJ in the root region. Histological analysis of control tooth root revealed a distinct and clearly visible cementum layer and a clearly delineated CDJ, while the HPP + HCH root demonstrated a notably reduced or absent cementum layer (Fig. 4U–X).

## Discussion

In this study, we have identified and characterized tooth phenotypic features in four patients with distinct skeletal disorders. The skeleton and teeth share commonalities in terms of developmental biology, structure, and mineral composition. In this study, the four patients exhibited common clinical characteristics such as short stature, bone deformities, and dental abnormalities.

LTBP3, a member of the latent TGF-β binding protein family, possesses multiple domains, including a TGF-β-binding domain, epidermal growth factor (EGF) domains, and calcium-binding EGF domains [27]. *Ltbp3* is expressed in multiple organs during development, including teeth, bones, brain, and the cardiac outflow tract [28]. Monoallelic variants in *LTBP3* lead to ACMICD and geleophysic dysplasia, resulting in a disproportionate short stature with a short trunk, distinctive facial features, and heart diseases, and an increased susceptibility to thoracic aortic aneurysms and dissections. On the other hand, biallelic variants in *LTBP3* cause dental anomalies (amelogenesis imperfecta) and short stature. Patient-1 was diagnosed with ACMICD harboring the heterozygous p.(Gly673Cys) mutation in *LTBP3*, which is predicted to induce alterations in LTBP3’s configuration and function [20]. The ACMICD tooth sample exhibited a significant reduction in microhardness in both enamel and dentin, along with a lower Ca/P ratio in dentin compared to controls. Furthermore, the enamel of ACMICD patient exhibited deeper grooves surrounding the enamel prism when compared to controls. These findings align with a previous study demonstrating that *Ltbp3*^*−/−*^ mice displayed impaired enamel mineralization, disrupted formation of enamel nodules, and an altered enamel prism pattern [29]. Collectively, these data indicate the importance of LTBP3 in tooth mineralization processes.

FGFR3 is a regulatory molecule that plays a crucial role in various biological processes, such as endochondral ossification, chondrogenesis, mitogenesis, angiogenesis, and wound healing [30]. Pathogenic variants in *FGFR3* are associated with several skeletal disorders, including ACH, HPP, and thanatophoric dysplasia. ACH is the most prevalent SDs characterized by a severe and disproportionate short stature, frontal bossing, and midfacial hypoplasia. The p.Gly380Arg gain-of-function mutation in *FGFR3* accounts for approximately 97% of ACH cases, leading to impaired bone ossification and reduced bone size, particularly in long bones. Notably, more than 80% of ACH patients exhibit de novo mutations in *FGFR3* [30]. As anticipated, patient-4, who exhibited characteristic features of ACH such as short stature, relative macrocephaly, and maxillary hypoplasia, was found to harbor the commonly reported de novo mutation, p.Gly380Arg, in the *FGFR3* gene. This mutation is well documented in ACH cases and is known to contribute to the pathogenesis of the disorder. To date, there have been no prior studies investigating the physical properties and ultrastructures of teeth in mice or humans with *FGFR3* mutations. However, it is known that *FGFR3* is expressed prominently in the developing dental epithelium and mesenchyme, particularly in the odontoblast layer [31]. Accordingly, our study observed a significant reduction in microhardness of the ACH dentin. However, the microhardness and mineral content of ACH enamel were found to be comparable to those of the controls. These findings suggest that FGFR3 likely plays a role in dentin formation, and alterations in FGFR3 function could potentially lead to clinically imperceptible changes in dentin composition.

Approximately 70% of HCH patients carry the heterozygous p.Asn540Lys mutation in *FGFR3*. The skeletal characteristics of HCH closely resemble those of ACH, but their severity tends to be milder due to the weakly overactivated function of FGFR3 [32]. The *ALPL* gene, on the other hand, encodes tissue-nonspecific alkaline phosphatase, an enzyme that plays a crucial role in the regulation of bone and tooth mineralization [33]. Homozygous or compound heterozygous mutations in *ALPL* lead to HPP, a condition characterized by impaired mineralization of growing or remodeling bone. The severity of HPP can vary, ranging from a severe perinatal form with pulmonary insufficiency and absence of mineralized bone to a milder form known as odontohypophosphatasia. In odontohypophosphatasia, patients experience premature loss of teeth with intact roots, but do not exhibit any skeletal malformations [34]. Patients affected with HPP displayed impaired cementum formation, similar to tooth features in mice lacking TNAP activity [8, 35, 36]. Patient-2, who presented with a combination of HPP and HCH along with mutations in *ALPL* and *FGFR3*, exhibited typical dental characteristics of HPP. These included a deficient cementum layer, premature loss of primary tooth with non-resorbed root, an enlarged pulp cavity with thin dentinal walls, and abnormalities in dentin mineral composition. The observed dentin abnormalities in patient-2 align with previous research finding reporting defective dentin mineralization in mice with *Alpl* mutations [37]. TNAP is expressed in various stem cells derived from the dental pulp, periodontal ligament, and bones [38, 39]. Studies have reported that FGF can inhibit alkaline phosphatase activity, leading to reduced mineralization in dental stem cells and osteoblasts [40–42]. In the patient with HPP + HCH, it is plausible that the gain-of-function mutation in *FGFR3* could potentially impair the functions of ALPL and TNAP, resulting in significantly lower Ca/P ratios in both enamel and dentin. However, these specific alterations were not observed in the patient with ACH (*FGFR3* mutation) or reported in individuals with HPP (*ALPL* mutation) [8].

*RUNX2* encodes the runt-related transcription factor, which plays a critical role in the development and maintenance of teeth, bones, and cartilage [43]. The heterozygous mutations in *RUNX2* lead to CCD, characterized by wide skull sutures, clavicular hypoplasia, short stature, and multiple supernumerary teeth. *RUNX2* is expressed in various dental tissues, including the osteogenic mesenchyme of the developing maxilla and mandible, dental papilla, ameloblasts, odontoblasts, and dental pulp cells [44]. Previous research has demonstrated that the dentin in an individual with CCD exhibits a flattened DEJ, irregular dentinal tubules, and irregular intertubular dentin, along with significantly decreased levels of calcium and phosphorus and increased oxygen content [45]. In line with these findings, the tooth obtained from patient-3, who carried the *RUNX2* p.(Arg225Trp) mutation, demonstrated a substantial decrease in Ca/P level, which correlated with a significant reduction in enamel microhardness. In addition, we observed a flatter DEJ and obscure intertubular dentin, indicative of inadequate mineralization, in the CCD tooth. These observations align with a study that reported a significant reduction in enamel mineralization in *K14-Cre;Runx2*^*flox/flox*^ mice [46]. Collectively, our findings provide further confirmation that *RUNX2* is involved in the mineralization process and influences the physical properties of both enamel and dentin [2].

While alterations in the Ca/P ratio provide insights into changes in the main mineral components of dental hard tissues, they may not fully reflect their microhardness. Other minerals, including carbon, nitrogen, oxygen, sodium, and magnesium, present in enamel and dentin also contribute to their hardness [47]. These additional elements might account for the inconsistent relationship observed between microhardness and Ca/P ratio in enamel and/or dentin of teeth affected by different SDs. Interestingly, in all affected teeth, the lingual surfaces were significantly smoother, while the buccal surfaces were significantly rougher compared to controls, except for the buccal surface of ACMICD tooth, which did not show significant differences from controls. During tooth development, odontogenic genes exhibit time- and location-specific expression patterns. For example, *Runx1* is expressed in the lingual side of the outer dental epithelium during the bell stage [48], and *Sox2*, *Sox6*, and *Sox13* are expressed in the lingual bud epithelium [49]. Moreover, *Spry2*^+*/−*^;*Spry4*^*−/−*^ mice, which display upregulated FGF signaling, exhibit ectopic enamel formation on the lingual side of the teeth [50]. These findings suggest that there is differential gene expression between different tooth surfaces, and genetic alterations in genes such as *FGFR3*, *RUNX2*, *LTBP3*, and *ALPL*, which are involved in tooth and bone development, may contribute to the formation of tooth structure. However, further investigations are required to fully understand the molecular mechanisms underlying the differences in tooth characteristics between patients with SDs and healthy individuals.

The changes in tooth characteristics including surface roughness, microhardness, and mineral composition observed in patients affected by SDs can significantly impact the prognosis and response to dental treatment. Teeth with reduced hardness are more susceptible to wear and fracture. Abnormal properties and composition of enamel and dentin can compromise the effectiveness of resin adhesives used in treatments. Incomplete formation of the hybrid layer due to limited resin penetration can lead to microleakage, enzymatic degradation, and long-term treatment failures [51]. Additionally, defects in cementum, as observed in HPP, contribute to premature tooth loss. It has also been reported that teeth with defective dentin due to genetic disorders are at a higher risk of periapical infections [52, 53]. Consequently, early detection of underlying genetic diseases enables dentists and healthcare professionals to develop appropriate treatment plans as part of precision dental medicine. However, it should be noted that due to the rarity of SDs and the limitation of obtaining only a single tooth per case, our study may not capture all possible characteristics of these teeth, and there may be variances that were undetected. Therefore, future studies with larger sample sizes of patients with SDs are necessary to confirm and expand upon our findings, providing a better understanding of the dental characteristics associated with these disorders.

## Conclusions

This study represents the first comprehensive investigation into the changes in tooth characteristics, encompassing physical properties, mineral content, and ultrastructure, in patients affected by SDs. By elucidating these significant findings, our study expands the understanding of the phenotypic spectra associated with SDs. This, in turn, paves the way for further research into genotype–phenotype correlations and enables more precise dental management strategies for individuals affected by these conditions.

### Supplementary Information

Below is the link to the electronic supplementary material.

Supplementary file1 (PDF 97 KB)

## Data Availability

All data supporting the findings of this study are available within the paper and its Supplementary Information.

## References

[1] Krakow D, Rimoin DL (2010). The skeletal dysplasias. Genet Med.

[2] Camilleri S, McDonald F (2006). Runx2 and dental development. Eur J Oral Sci.

[3] Thaweesapphithak S, Saengsin J, Kamolvisit W (2022). Cleidocranial dysplasia and novel RUNX2 variants: dental, craniofacial, and osseous manifestations. J Appl Oral Sci.

[4] Xue Y, Sun A, Mekikian PB (2014). FGFR3 mutation frequency in 324 cases from the International Skeletal Dysplasia Registry. Mol Genet Genomic Med.

[5] Al-Saleem A, Al-Jobair A (2010). Achondroplasia: craniofacial manifestations and considerations in dental management. Saudi Dent J.

[6] Choi TM, Kramer GJC, Goos JAC (2022). Evaluation of dental maturity in Muenke syndrome, Saethre-Chotzen syndrome, and TCF12-related craniosynostosis. Eur J Orthod.

[7] Huckert M, Stoetzel C, Morkmued S (2015). Mutations in the latent TGF-beta binding protein 3 (LTBP3) gene cause brachyolmia with amelogenesis imperfecta. Hum Mol Genet.

[8] van den Bos T, Handoko G, Niehof A (2005). Cementum and dentin in hypophosphatasia. J Dent Res.

[9] Kramer K, Chavez MB, Tran AT (2021). Dental defects in the primary dentition associated with hypophosphatasia from biallelic ALPL mutations. Bone.

[10] Dard M (1993). Histology of alveolar bone and primary tooth roots in a case of cleidocranial dysplasia. Bull Group Int Rech Sci Stomatol Odontol.

[11] Intarak N, Budsamongkol T, Theerapanon T (2021). Tooth ultrastructure of a novel COL1A2 mutation expanding its genotypic and phenotypic spectra. Oral Dis.

[12] Budsamongkol T, Intarak N, Theerapanon T (2019). A novel mutation in COL1A2 leads to osteogenesis imperfecta/Ehlers-Danlos overlap syndrome with brachydactyly. Genes Dis.

[13] Hemwong N, Phokaew C, Srichomthong C (2020). A patient with combined pituitary hormone deficiency and osteogenesis imperfecta associated with mutations in LHX4 and COL1A2. J Adv Res.

[14] Udomchaiprasertkul W, Kuptanon C, Porntaveetus T (2020). A family with homozygous and heterozygous p.Gly337Ser mutations in COL1A2. Eur J Med Genet.

[15] Craig RG, Peyton FA (1958). The micro-hardness of enamel and dentin. J Dent Res.

[16] Caldwell RC, Muntz ML, Gilmore RW (1957). Microhardness studies of intact surface enamel. J Dent Res.

[17] Pilar G-S, Reyes-Gasga J (2003) Microhardness and chemical composition of human tooth. Mater Res 6. 10.1590/S1516-14392003000300011

[18] Teutle-Coyotecatl B, Contreras-Bulnes R, Rodriguez-Vilchis LE et al (2022) Effect of surface roughness of deciduous and permanent tooth enamel on bacterial adhesion. Microorganisms 10. 10.3390/microorganisms10091701 10.3390/microorganisms10091701 PMC9501044 36144302

[19] De Menezes Oliveira MA, Torres CP, Gomes-Silva JM (2010). Microstructure and mineral composition of dental enamel of permanent and deciduous teeth. Microsc Res Tech.

[20] Intarak N, Theerapanon T, Thaweesapphithak S (2019). Genotype-phenotype correlation and expansion of orodental anomalies in LTBP3-related disorders. Mol Genet Genomics.

[21] Porntaveetus T, Srichomthong C, Suphapeetiporn K (2017). Monoallelic FGFR3 and biallelic ALPL mutations in a Thai girl with hypochondroplasia and hypophosphatasia. Am J Med Genet A.

[22] Jaruga A, Hordyjewska E, Kandzierski G (2016). Cleidocranial dysplasia and RUNX2-clinical phenotype–genotype correlation. Clin Genet.

[23] Quack I, Vonderstrass B, Stock M (1999). Mutation analysis of core binding factor A1 in patients with cleidocranial dysplasia. Am J Hum Genet.

[24] Cohen MM (2009). Perspectives on RUNX genes: an update. Am J Med Genet Part A.

[25] Vajo Z, Francomano CA, Wilkin DJ (2000). The molecular and genetic basis of fibroblast growth factor receptor 3 disorders: the achondroplasia family of skeletal dysplasias, Muenke craniosynostosis, and Crouzon syndrome with acanthosis nigricans*. Endocr Rev.

[26] Placone J, Hristova K (2012). Direct assessment of the effect of the Gly380Arg achondroplasia mutation on FGFR3 dimerization using quantitative imaging FRET. PLOS One.

[27] Robertson IB, Horiguchi M, Zilberberg L (2015). Latent TGF-β-binding proteins. Matrix Biol.

[28] Huckert M, Stoetzel C, Morkmued S (2015). Mutations in the latent TGF-beta binding protein 3 (LTBP3) gene cause brachyolmia with amelogenesis imperfecta. Hum Mol Genet.

[29] Morkmued S, Hemmerle J, Mathieu E (2017). Enamel and dental anomalies in latent-transforming growth factor beta-binding protein 3 mutant mice. Eur J Oral Sci.

[30] Ornitz DM, Marie PJ (2019). Chapter eight - fibroblast growth factors in skeletal development. Curr Top Dev Biol.

[31] Guo W, Lin X, Zhang R (2022). Spatiotemporal expression patterns of critical genes involved in FGF signaling during morphogenesis and odontogenesis of deciduous molars in miniature pigs. Int J Med Sci.

[32] Bober M, Bellus GA, Nikkel SM, Tiller GE (2020) Hypochondroplasia. In: GeneReviews®. Adam MP, Mirzaa GM, Pagon RA, Wallace SE, Bean LJH, Gripp KW, Amemiya A (eds) University of Washington, Seattle (WA) 20301650

[33] Hu JC, Plaetke R, Mornet E (2000). Characterization of a family with dominant hypophosphatasia. Eur J Oral Sci.

[34] Whyte MP (2016). Hypophosphatasia — aetiology, nosology, pathogenesis, diagnosis and treatment. Nat Rev Endocrinol.

[35] Beertsen W, VandenBos T, Everts V (1999). Root development in mice lacking functional tissue non-specific alkaline phosphatase gene: inhibition of acellular cementum formation. J Dent Res.

[36] Reibel A, Maniere MC, Clauss F (2009). Orodental phenotype and genotype findings in all subtypes of hypophosphatasia. Orphanet J Rare Dis.

[37] Foster BL, Nagatomo KJ, Tso HW (2013). Tooth root dentin mineralization defects in a mouse model of hypophosphatasia. J Bone Miner Res.

[38] Tomlinson MJ, Dennis C, Yang XB (2015). Tissue non-specific alkaline phosphatase production by human dental pulp stromal cells is enhanced by high density cell culture. Cell Tissue Res.

[39] Melms H, Herrmann M, Förstner K (2020). Novel molecular cues for dental defects in hypophosphatasia. Exp Cell Res.

[40] Chang M-C, Chen N-Y, Chen J-H (2021). bFGF stimulated plasminogen activation factors, but inhibited alkaline phosphatase and SPARC in stem cells from apical papilla: involvement of MEK/ERK, TAK1 and p38 signaling. J Adv Res.

[41] Nowwarote N, Sukarawan W, Pavasant P (2018). Basic fibroblast growth factor regulates phosphate/pyrophosphate regulatory genes in stem cells isolated from human exfoliated deciduous teeth. Stem Cell Res Ther.

[42] Mansukhani A, Bellosta P, Sahni M (2000). Signaling by fibroblast growth factors (FGF) and fibroblast growth factor receptor 2 (FGFR2)-activating mutations blocks mineralization and induces apoptosis in osteoblasts. J Cell Biol.

[43] Komori T (2002). Runx2, a multifunctional transcription factor in skeletal development. J Cell Biochem.

[44] Chen S, Gluhak-Heinrich J, Wang YH (2009). Runx2, osx, and dspp in tooth development. J Dent Res.

[45] Xuan D, Sun X, Yan Y (2010). Effect of cleidocranial dysplasia-related novel mutation of RUNX2 on characteristics of dental pulp cells and tooth development. J Cell Biochem.

[46] Chu Q, Gao Y, Gao X (2018). Ablation of Runx2 in ameloblasts suppresses enamel maturation in tooth development. Sci Rep.

[47] Manoor A, Moeen F, Mehmood M (2019). Correelation between micro-hardness and mineral content in the healthy tooth enamel of humans belonging to different age groups. PAFMJ.

[48] Yamashiro T, Åberg T, Levanon D (2002). Expression of Runx1, -2 and -3 during tooth, palate and craniofacial bone development. Mech Dev.

[49] Kawasaki K, Kawasaki M, Watanabe M (2015). Expression of Sox genes in tooth development. Int J Dev Biol.

[50] Klein OD, Lyons DB, Balooch G (2008). An FGF signaling loop sustains the generation of differentiated progeny from stem cells in mouse incisors. Development.

[51] Betancourt DE, Baldion PA, Castellanos JE (2019). Resin-dentin bonding interface: mechanisms of degradation and strategies for stabilization of the hybrid layer. Int J Biomater.

[52] Porntaveetus T, Nowwarote N, Osathanon T (2019). Compromised alveolar bone cells in a patient with dentinogenesis imperfecta caused by DSPP mutation. Clin Oral Investig.

[53] Porntaveetus T, Osathanon T, Nowwarote N (2018). Dental properties, ultrastructure, and pulp cells associated with a novel DSPP mutation. Oral Dis.

