# Supplementary material for: Dental characteristics of patients with four different types of skeletal dysplasias
Source: Clin Oral Investig. 2023 Aug 7;27(10):5827–39. doi: 10.1007/s00784-023-05194-w (PMC10560164; doi:10.1007/s00784-023-05194-w)
Supplement: Supplementary file 1 — Supplementary file1 (PDF 97 KB) [file 784_2023_5194_MOESM1_ESM.pdf]

Supplementary Data

Dental characteristics of patients with four different types of skeletal dysplasias

Worasap Tantibhaedhyangkul[1,2], Jenjira Tantrapornpong[1], Nuttanun Yutchawit[1], Thanakorn Theerapanon[1], Narin Intarak[1], Sermporn Thaweesapphithak[1], Thantrira Porntaveetus[1,*], Vorasuk Shotelersuk[3,4]

[1]Center of Excellence in Genomics and Precision Dentistry, Department of Physiology, Faculty of Dentistry, Chulalongkorn University, Bangkok, 10330, Thailand

[2]Department of Prosthodontics, Faculty of Dentistry, Chulalongkorn University, Bangkok, 10330, Thailand

[3]Center of Excellence for Medical Genomics, Medical Genomics Cluster, Department of Pediatrics, Faculty of Medicine, Chulalongkorn University, Bangkok, 10330, Thailand

[4]Excellence Center for Genomics and Precision Medicine, King Chulalongkorn Memorial Hospital, the Thai Red Cross Society, Bangkok, 10330, Thailand

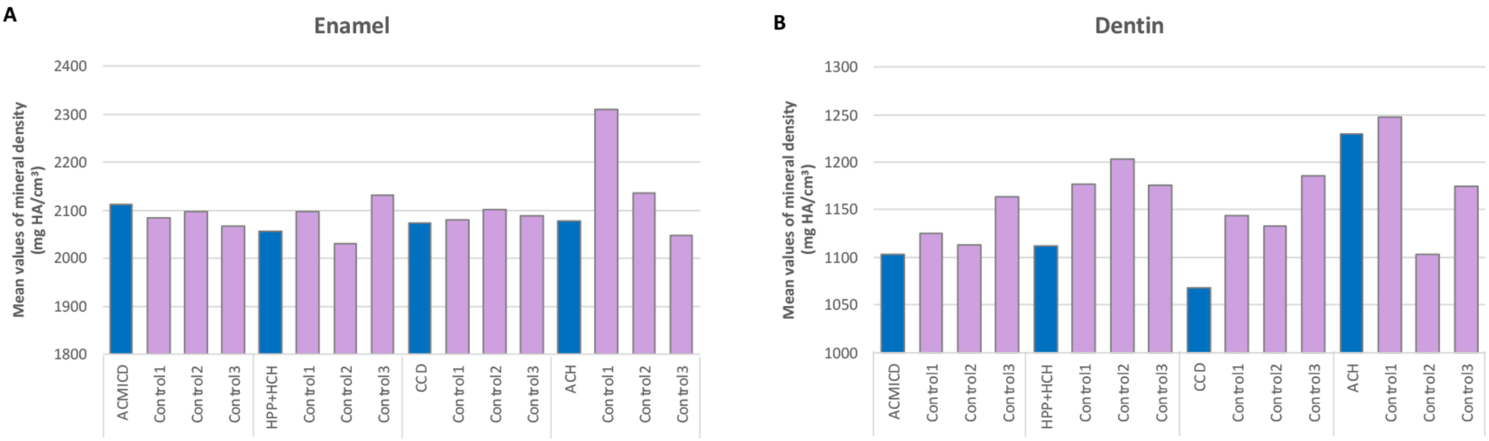

Figure S1. Mean mineral density values of skeletal dysplasia teeth and controls. The skeletal dysplasia teeth did not show significant differences in the mean mineral density values in the enamel (A) and dentin (B), compared with those in controls. ACMICD, acromicric dysplasia, HPP, hypophosphatasia, HCH, hypochondroplasia, CCD, cleidocranial dysplasia; ACH, achondroplasia.

Table S1. Mineral density of teeth associated with skeletal dysplasia

Samples	Mean mineral density values (mg HA/cm^3)	
	Enamel	Dentin
**ACMICD**	2112.0840	1102.9993
Control1	2084.0576	1124.8339
Control2	2097.3752	1112.8022
Control3	2066.6072	1163.8678
**HPP+HCH**	2057.3460	1112.0470
Control1	2097.7424	1177.2770
Control2	2029.9612	1203.8202
Control3	2132.3510	1176.1040
**CCD**	2074.0466	1067.5229
Control1	2081.2104	1144.2094
Control2	2101.3245	1132.6406
Control3	2089.1091	1185.3594
**ACH**	2078.3633	1229.7203
Control1	2311.5560	1246.8953
Control2	2135.9497	1103.5260
Control3	2047.5955	1174.8892

ACMICD, acromicric dysplasia, HPP, hypophosphatasia, HCH, hypochondroplasia, CCD, cleidocranial dysplasia; ACH, achondroplasia.
